# Percutaneous calcaneoplasty in displaced intraarticular calcaneal fractures

**DOI:** 10.1007/s10195-013-0249-8

**Published:** 2013-06-08

**Authors:** Francesco Biggi, Stefano Di Fabio, Corrado D’Antimo, Francesco Isoni, Cosimo Salfi, Silvia Trevisani

**Affiliations:** Orthopaedics and Traumatology Department, San Martino Hospital, Viale Europa 22, 32100 Belluno, Italy

**Keywords:** Calcaneus, Fracture, Minimally-invasive, Balloon reduction

## Abstract

The ideal treatment for displaced intraarticular calcaneal fractures is still under debate. Open reduction and internal fixation is the most popular surgical procedure; however, wound complications, hardware failure and infection remain a major concern. The aim of this study was to evaluate the results of a new minimally-invasive surgical procedure: closed reduction technique combined with balloon-assisted fracture augmentation with cement or calcium phosphate (minimally-invasive percutaneous calcaneoplasty). We retrospectively reviewed 11 patients that sustained Sander’s type II and III calcaneal fractures treated in our institution from January 2008 to June 2010. The same approach and technique was utilized in all cases. Conventional X-rays and CT scan have been performed pre- and post-operatively. The average follow-up was 24 months. The American Orthopaedic Foot and Ankle Society ankle/hindfoot score has been utilized for clinical evaluation and Bohler’s angle to assess bone reduction. All cases obtained bony union in 2/3 months, with average Bohler’s angle of 22.97° (from 14.21° to 32.83°). No skin complications or adverse reactions were observed, with only one patient complaining of residual pain in the hindfoot. Minimally-invasive percutaneous calcaneoplasty can represent an alternative to open reduction internal fixation in the treatment of calcaneal fractures, allowing stable reduction without plating, early function recovery and short hospital stay.

## Introduction

Calcaneus is the most frequently fractured tarsal bone, accounting for about 2 % of all fractures, often derived from high-energy trauma in young patients.

The ideal treatment for displaced intraarticular calcaneal fractures remains controversial. Nevertheless, there is evidence from studies with large patient cohorts that fragment reduction, with anatomical Bohler’s angle restoration, and subtalar joint congruity predict higher functional scores as well as a lower incidence of post-traumatic arthritis [1, 2]. Open reduction internal fixation (ORIF) is the most popular surgical approach, utilizing a lateral approach to expose fragments, obtain reduction, and stabilize by plating with additional bone grafting [1]. However, soft tissue complications remain a major concern, due to the thin and vulnerable skin over the lateral calcaneal wall, which is cut and retracted during surgery, and jeopardized by the underneath plate. The reported rate of complications is reported between 15 and 25 %, with additional problems arising from delayed work recovery and compensation [3, 4].

Looking at our belief in terms of minimally-invasive percutaneous osteosynthesis (MIPO), and supported by direct experience in kyphoplasty techniques for compression vertebral fractures, we started in 2008 the application of Kyphon (Medtronic) tools, in association with minimally-invasive techniques, for closed reduction of Sander’s type II and III fractures and balloon-assisted augmentation with both acrylic cement and calcium phosphate (minimally-invasive percutaneous calcaneoplasty).

Our targets were to (1) minimize surgical trauma by reducing complications, (2) standardize the technique, (3) avoid immobilization by encouraging early function, and (4) allow partial to full weight-bearing in 4–6 weeks.

## Materials and methods

**Fig. 1:**
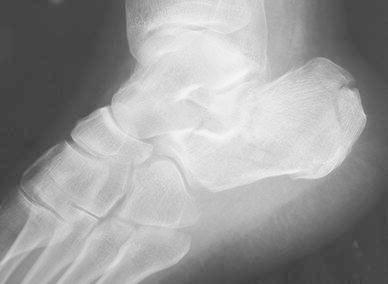
Lateral preoperative radiographs of a Sanders type III thalamic fracture

**Fig. 2:**
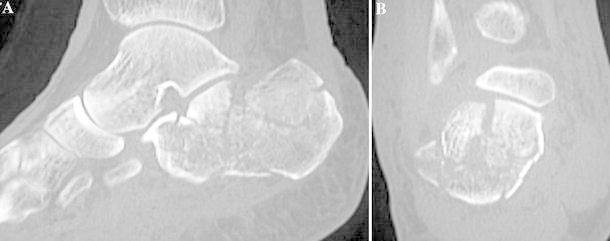
**a, b** Preoperative CT scan reconstruction showing *vertical* displacement of the thalamic joint surface

**Fig. 3:**
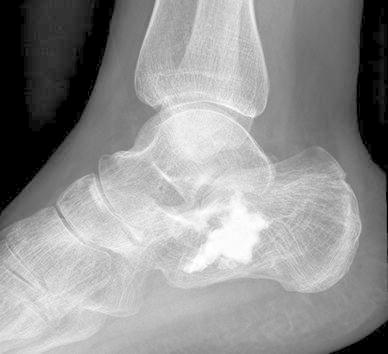
Four years postoperative lateral radiograph showing reconstruction and restoration of the thalamic surface

We retrospectively reviewed 11 patients, seven female and four male, with a mean age of 58.4 years (from 28 to 81 years) who sustained displaced intraarticular calcaneal fractures and were treated with minimally-invasive percutaneous calcaneoplasty at our institution in the period from January 2008 to June 2010. Preoperatively, conventional X-rays and CT scan with 2D and 3D reconstructions were obtained in all cases (Figs. 1, 2a, b), and surgery was scheduled in 2–3 days. A total of six fractures were classified as Sanders type II, five as type III. The main injury mechanism was a fall from height on hard ground in seven of the cases (64 %), followed by motor vehicle accidents in the remaining three (27 %). Conventional X-rays were performed postoperatively and at 6–8 weeks, together with a CT scan when usually full weight-bearing is allowed, at 1 year, and at the last follow-up (Fig. 3).

The American Orthopaedic Foot and Ankle Society (AOFAS) ankle/hindfoot score was utilized for clinical evaluation, grading results as excellent (90), good (80), fair (70) and poor (<70); Bohler’s angle was calculated to assess bone reduction. Time requested to regain normal daily activity was also scheduled.

### Operative technique

**Fig. 4:**
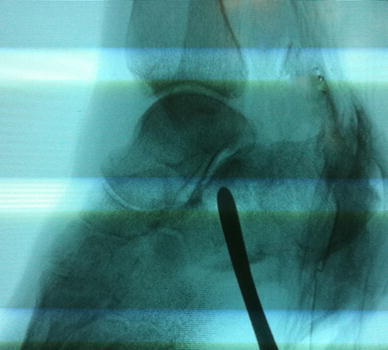
Reduction of subtalar joint with periosteum elevator under image intensifier control

**Fig. 5:**
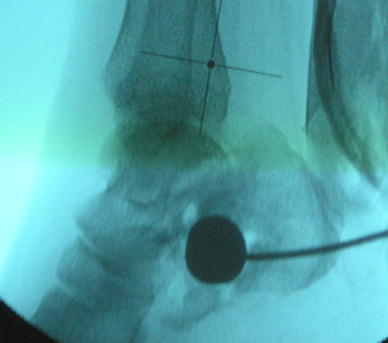
Intraoperative view demonstrating position of the balloon

A 2-cm incision is located on the lateral-posterior calcaneal wall, and the sinus tarsi is approached by visualizing the depressed articular surface and relative numbers of fragments: usually a smooth periosteum elevator is utilized, under image intensifier control, to lift and relocate the articular process (Fig. 4) temporary stabilized with K-wires. With an axial view, if a usually varus malalignment is present, a single manipulation or a combined one with a Steinman pin insertion in the tuber perpendicular to the longitudinal axis of the calcaneus can help in solving the problem. Then a cannula is placed into the calcaneus body followed by insertion of a bone tamp attached to a digital manometer (Kyphon^®^, Medtronic, Minneapolis, MN, USA). The balloon is inflated gradually under fluoroscopy (Fig. 5); at this time the bone cement was prepared immediately prior to its injection into the defect and the balloon was removed. No cast is applied, and patients encouraged to actively bend the ankle, with assisted weight-bearing and discharge in 2–3 days.

## Results

All cases progressed to bony union in 2–3 months. According to AOFAS score, clinical results were excellent in six cases, good in four and fair in one due to subtalar arthritic changes.

Considering a normal Bohler’s angle between 20° and 40°, the mean preoperative value was 9.91° (6.15°–18.78°), while the mean postoperative was 22.97° (14.21°–32.83°) (Table 1). Patients returned to work between 10 and 12 weeks. No wound complications or adverse reactions were observed.

**Table 1:** Patients data

Patient	Sex	Age	Sanders type	Augmentation	AOFAS score	Preop. Bohler’s angle	Postop. Bohler’s angle	Follow-up (months)
1	M	81	III	PMMA	87	9.23°	21.54°	31
2	F	71	II	PMMA	93	10.64°	20.78°	42
3	F	62	III	PMMA	73	6.15°	14.21°	15
4	F	72	II	PMMA	88	9.47°	23.89°	28
5	M	28	II	CP	94	11.21°	27.21°	31
6	F	52	III	PMMA	86	8.16°	19.91°	29
7	F	57	II	PMMA	95	18.78°	32.83°	21
8	M	48	III	CP	93	10.58°	26.67°	24
9	M	61	II	PMMA	91	9.37°	23.14°	16
10	F	57	III	PMMA	82	7.51°	19.73°	14
11	F	53	II	PMMA	92	7.96°	22.74°	13
Average (± D)		58.4 (±14.1)			88.5 (±6.5)	9.91 (±3.30)	22.97 (±4.83)	24 (±9)

*PMMA* polymethylmethacrylate, *CP* calcium phosphate, *AOFAS* American Orthopaedic Foot and Ankle Society (the score was calculated at latest follow-up visit)

## Discussion

Calcaneal morphology and height are essential for hindfoot and ankle function, and fracture reduction, recreating a congruent subtalar joint, is mandatory.

Different techniques, both conservative or operative, have been proposed for the treatment of displaced intraarticular calcaneal fractures. ORIF, via the extended lateral L-type retro-malleolar approach and plate fixation, is still the most popular method, reporting good to excellent results in 60–85 % of cases [1–3].

In general, young active patients with displaced but reconstructable articular fractures should be considered for surgical intervention, trying to obtain the best possible anatomical reconstruction, necessary for a better functional outcome. Normally, surgical treatment is delayed until soft tissue swelling subsided, and a wide range of percutaneous or less invasive techniques have been described.

Percutaneous kyphoplasty using a balloon has been used, in recent years, in spine surgery to treat osteoporotic and acute vertebral fractures [5, 6]: the balloon is utilized to provide upward lifting of the vertebral endplate in a body compression fracture, allowing injection of cement or calcium phosphate to provide stability. We applied the same technique and tools to treat displaced but reconstructable articular calcaneus fractures, utilizing a minimally invasive percutaneous lateral approach, obtaining reduction by manipulating fragments under fluoroscopy, and finally stabilizing the subtalar surface by injecting cement or a more biologic but resistant material (calcium phosphate). Literature about this new minimally-invasive technique is very poor [7–9]. Gupta et al. [7] and Jacquot and Atchabahian [8] reported excellent outcome in terms of bone healing and complications rate by treating calcaneal fractures with the balloon. However, those studies presented a small cohort of patients with no statistical analysis. Broome et al. [10] reported a cadaveric study on utilizing balloon for reducing intraarticular fractures of the tibial plateau and distal radius: the inflatable tamp was successful in reducing all fractures without complications.

Despite the limited number of cases with a minimum 1 year follow-up, the results are very encouraging, offering a true minimally-invasive percutaneous stabilization as an alternative in the surgical treatment of such difficult fractures.

Additional cases and longer follow-up are needed to support these data.
